# Policy Announcement, Investor Attention, and Stock Volatility: Evidence From the New Energy Vehicle Industry

**DOI:** 10.3389/fpsyg.2022.838588

**Published:** 2022-02-23

**Authors:** Mimi Su, Chen Wang

**Affiliations:** ^1^School of Economics, Shandong University, Jinan, China; ^2^School of Statistics, Shandong University of Finance and Economics, Jinan, China

**Keywords:** new energy vehicle, policy announcement, stock volatility, investor attention, Chinese

## Abstract

New energy vehicle (NEV) policies have greatly promoted the growth of the NEV industry in China, while also attracting a lot of investor attention. Using Chinese NEV concept stocks and related industrial policies, including purchase tax incentives (PTI) and promotion and application (P&A) policies, issued from 2011 to 2020 as the research setting, this paper adopts a panel data model to examine the impact of policy announcement on the volatility of NEV concept stocks, as well as the mediating role of investor attention in transmitting the impact. We find that NEV P&A policies have a significant and positive impact on NEV concept stock volatility, while PTI policies do not have a significant impact. Moreover, investor attention plays a partial mediating role in transmitting the impact of P&A policies on NEV stock market by increasing the stock volatility risk. Furthermore, there is heterogeneous effect of equity ownership in the relationship between policy announcement and investor attention on the volatility of NEV concept stocks; non-state-owned firms are more sensitive to the NEV P&A policies than state-owned firms. By analyzing the relationship between policy announcement and concept stock volatility, this paper enriches the research on NEV concept stocks and provides policy implications for the NEV industry.

## Introduction

As global warming intensifies, countries have introduced a variety of carbon emission reduction policies to promote sustainable development (Yang et al., 2020). With the transportation sector being one of the major sources of carbon emissions (Zhu and Li, 2017), the development of new energy vehicles (NEV) is thus critical to building a low-carbon society (Liu et al., 2021). From 2001 to 2020, the Chinese government implemented a series of policies to encourage the use of NEV (Tian et al., 2021; Wan et al., 2021), including regulations, financial incentives, pilot demonstrations, and charging infrastructure deployment (Tian et al., 2020). These policies, together with energy market reform, have assisted the rapid growth of China’s NEV industry; in 2020, the sales of NEVs reached about 1.4 million units, accounting for roughly two fifths of global totals (Wu et al., 2021).

Policy announcement not only has an impact on the real economy, but they may also cause stock market volatility. Government control and regulation are essential for a functioning stock market, and government policies can play a regulatory role in stock markets. Previous studies have shown that macro policies have a significant impact on stock market volatility (Engle and Rangel, 2008). The socialist market economy with Chinese characteristics is a combination of effective government intervention and efficient market, and policy changes have a strong influence on Chinese economy (Xue et al., 2019, 2021). Compared to other countries, the Chinese government implements policies more frequently (Zhang et al., 2021a), and the impact of policy changes on Chinese stock market is more prominent (You et al., 2017). Therefore, it is important to explore the impact of policy changes on the stock market of NEVs in China. Concept stocks are a distinct category of the Chinese stock market; it is a collection of stocks related to a specific sector that have emerged in response to hot events or concepts. China’s NEV industry policies have given rise to the “NEV concept stock” segment in the stock market. On 23 April 2020, the day the NEV subsidy extension policy^1^ was released, the Baidu search volume for NEV stocks increased by 18% month on month.^2^

Different policies have different impact on the stock market. For example, monetary policies can cause larger stock market fluctuations compared to fiscal policies. Currently, NEV-related policies mainly focus on NEV purchase tax incentives (PTI) and promotion and application (P&A). Since there is a lack of comparative research on such policies in prior studies, this paper divides NEV policies into two categories, namely, purchase tax incentives (PTI) and P&A policies, and analyzes their impact on the volatility of related concept stocks.

Investor attention is an important cause of liquidity and volatility in stock markets (Fang and Peress, 2009). When faced with a large amount of information, investors may make inaccurate judgments due to their limited information processing ability and herd effects (Hirshleifer and Teoh, 2003; Barber and Odean, 2008), and they tend to be drawn to hot concepts or events and prefer to focus on hot markets rather than a specific stock (Carro et al., 2015; Balcilar et al., 2017). A concept stock is a type of stock that was created as result of a hot event or concept, and it is subject to the board effect, which means that stocks of the same concept rise and fall together. Therefore, this paper will investigate the mediating effect of investment attention in the impact of policy announcement on stock market volatility.

To sum up, China’s NEV policy has promoted the development of its NEV industry, which in turn attracts investor attention and causes stock market volatility. Given the sensitivity of the concept stock sector to policy changes and the differences in how different policies affect stock market, this paper conducts an empirical study on the impact of NEV policies on concept stock price behavior and the mediating effect of investor attention in conveying the impact of NEV policies into concept stocks. This paper takes Chinese NEV concept stocks as the research setting and selects relevant Chinese industrial policies, including PTI and P&A policies, issued from 2011 to 2020 for empirical analysis. We find that NEV P&A policies have a significant and positive impact on the volatility of NEV concept stocks, while the PTI do not have a significant impact. Moreover, increased investor attention will raise the impact of NEV P&A policies on NEV concept stock volatility. Furthermore, there is heterogeneity across natures of equity in the impact of policy announcement and investor attention on the volatility of NEV concept stocks—non-state-owned enterprises are more sensitive to NEV P&A policies than state-owned enterprises. By analyzing the relationship between policy announcement and the volatility of NEV concept stocks, this paper enriches the study of how policies affect NEV concept stocks. It also provides a theoretical reference for formulating effective NEV-related policies.

Using China’s concept stock market as the research setting, this paper investigates the impact of new energy policies on new energy-related stock price volatility and explores the potential channels through which policies affect the stock market by analyzing the mediating effect of investor attention. We believe that this will be a promising area for future research that can be extended to other developing-country stock market studies. First, we select concept stocks, a distinct segment of China’s stock market, as the research object to examine the impact of the announcement of NEV policies on the volatility of related stocks. Second, we investigate the relationship between investor attention and concept stock trading activity, providing new insights for future research on stock market volatility. More importantly, this paper examines the mediating effect of investor attention in the relationship between policy announcements and the stock market, adding to the body of literature on investor attention and providing references for future research.

The remainder of the paper is structured as follows. In the following section, we review the concept stocks and the literature related to the impact of policy announcement and investor attention on the stock market before proposing the research hypotheses. “Research Design” introduces the data sources and variables for the research design. “Results” conducts the empirical analysis and reports the results. “Conclusion” summarizes the findings and proposes policy recommendations.

## Literature Review

### Concept Stock

Concept stocks are created in response to certain events and are related to hot concepts of the stock market. Concept stocks have a greater advertising effect, because they not only represent a certain type of stock, but also signify popular market trends. Investors, especially retail investors, are more likely to engage in irrational investment behavior when a hot topic or concept is widely followed by the market, which boosts the popularity of concept stocks. In addition, concept stocks have a “board effect,” where stocks under the same concept share an internal consistency. For retail investors, who have limited information processing ability and are often unable to fully grasp the dynamics of each stock, they tend to follow the stock market hot spots and focus on a certain type of stock (Chen and Haga, 2021). As a result, investor attention swings to changing market hot topics, causing the corresponding concept stock price to rise and fall at the same time.

China’s NEV concept stock emerged in 2009, when the central government of China issued a policy on 23 January 2009, committing to vigorously developing the NEV industry, and proposing to implement energy-saving and NEV demonstration and promotion pilots in 13 cities, and providing purchase subsidies to enterprises that responded positively to the national policy. Since then, new energy concept stocks have been gaining popularity. China has been devoted to developing NEVs for the past 10 years (Zhang et al., 2021b), establishing a number of policies in the process, and NEV concept stocks have remained popular. In 2015, China issued the Notice on the Fiscal Support Policy for the Promotion and Application of NEVs from 2016 to 2020, which boosted the popularity of NEV concept stocks and enticed many car manufacturers to participate in the new energy transition. In 2020, the government further emphasized the need to accelerate the promotion of NEVs. Major companies saw this as an indication of a bright future for the NEV market and began to participate in its production, making NEV concept stocks surge in popularity.

### The Impact of Policy Announcement on Stock Markets

The development of stock markets is inseparable from government promotion and regulation; government policies can play a certain regulatory role in stock markets, and the state ensures the healthy and smooth operation of stock markets through various policies. Previous studies have found that stock market volatility is closely related to changes in macroeconomic indicators (Poon and Taylor, 1991), and the enactment of macroeconomic policies can lead to a significant increase in stock market volatility (Engle and Rangel, 2008). After the government announces a policy change, stock prices will react accordingly (Pastor and Veronesi, 2012). For example, Ramiah selected stocks listed on the Australian Stock Exchange from 2005 to 2011 as the research setting and assessed the impact of 19 environmental regulation announcements on stock prices using the event study method and found that the Australian stock market is more sensitive to announcements of carbon pollution reduction policies, which exhibit varying effects on abnormal stock returns (Ramiah et al., 2013).

This is especially true for the Chinese stock market, and in order to foster market growth, the government implemented various policies to intervene in the operation of the stock market, resulting in the formation of China’s “policy market.” The impact of policy announcement on Chinese stock market volatility is especially prominent in a bull market (You et al., 2017). The announcement of new environmental regulations will immediately have a negative impact on the stock returns of heavily polluting firms, and the market reaction will become more pronounced as the regulations are implemented (Guo et al., 2020). A new environmental inspection system has an even greater impact on the stock market than environmental regulations; it can result in a significant decrease in shareholder value for heavily polluting firms, and neither political connections nor large firm size can mitigate this effect (Sam and Zhang, 2020). New energy policies are no exception: Hsiao et al. (2021) find that solar energy policies announced between 2005 and 2020 not only increased the volatility of China’s domestic stock market but also had a significant impact on foreign markets in Japan, Germany, and the United States.

Therefore, the development of the stock market is inextricably linked to national policies. A large number of studies have explored the overall impact of macro policies on the stock market, and only a few scholars have analyzed the impact of a particular sector’s policies on its concept stocks. However, concept stocks, as a unique stock segment in China, are more sensitive to policy announcement due to their own characteristics. Current research in this area focuses on two perspectives: one is qualitative analysis, which quantitatively analyzes the impact of policy announcement on the stock market; the other is event study, which explores the impact of policies on relevant concept stocks before and after their promulgation. Prior research has confirmed a significant correlation between national policy announcement and relevant concept stocks in artificial intelligence, supply chain finance, and science and technology innovation (Tversky and Kahneman, 1973; He et al., 2011). Therefore, we argue that the promulgation of NEV policy will also have a significant impact on the volatility of related concept stocks, and we hypothesize as follows.

*Hypothesis 1*: There is a significant correlation between the promulgation of new energy vehicle policy and the volatility of new energy vehicle concept stocks.

Although policy announcement can have an impact on the stock market, the diversity of policy types leads to differences in their impact. For example, both monetary and fiscal policies can have an impact on the stock market, but in terms of volatility, the implementation of monetary policies is more likely to cause stock market volatility in most sectors (He et al., 2011). In addition, the impact varies depending on the effective duration of the policy: short-term policies have a greater impact on the stock market than medium- and long-term policies, and although medium- and long-term policies have a positive relationship with market volatility, they have a weaker impact on it (Foresti and Napolitano, 2017). The current NEV policies can be divided into two categories, purchase tax incentives (PTI) and P&A policies. Among them, the PTI policies are promulgated at lengthy intervals, with insufficient and tiny tax adjustments, and in a unitary manner, which cannot clearly reflect the strong support for NEVs in national policy. As the government continues to promote NEVs, the continuity of the P&A policies is constantly strengthened, and its stability is significantly improved. Thus, it can be adjusted according to market changes in a timely manner.

Therefore, this paper divides NEV policies into two categories by content, purchase tax incentives and P&A policies, examines their respective effects on the volatility of related concept stocks, and further explores the impact of different policy types on concept stocks. Hereby, we propose the following hypotheses.

*Hypothesis 1a*: Purchase tax incentives have no significant effect on the volatility of new energy vehicle concept stocks.

*Hypothesis 1b*: New energy vehicle promotion and application policies have a significant and positive effect on the volatility of new energy vehicle concept stocks.

### The Mediating Role of Investor Attention

Prior studies have discovered many modern financial anomalies that cannot be explained, such as Monday effect, equity premium puzzle, and media effect. To explain these anomalies, behavioral finance was born. Behavioral finance theory argues from a psychological perspective that investors’ behavioral decision making is complex that investors’ decisions are not always rational; on the other hand, it suggests that even if investors are rational, they are boundedly rational or of limited rationality (Barberis and Thaler, 2005). In behavioral finance, the easiest way to examine whether investors behave irrationally is to study investor behavior in real market conditions (Cooper and Kovacic, 2012). Previous research argues that people have limited processing ability when making investment decisions (Simon, 1955; Tversky and Kahneman, 1973). This is referred to as the limited attention theory, which is widely used in financial market research. Investor attention was thought to be an important cause of stock market liquidity and volatility and financial market anomalies (Fang and Peress, 2009).

Investor decision-making behavior will change as a result of their limited ability to allocate their attention, and process and interpret information (Hirshleifer and Teoh, 2003). Stocks that rise faster tend to attract the greatest attention and investment (Barber and Odean, 2008; Aboody et al., 2010), leading to higher stock returns and volatility (Seasholes and Wu, 2007). Therefore, when positive news was reported during the period of low investor attention, it helps to attract investor attention and affects the firm’s economic performance (Loh, 2010). Meanwhile, there are differences in the impact of investor attention on the stock market in different market dynamics; investors’ ability to interpret information is generally better in bull market than in the bear ones (Aouadi et al., 2018).

In addition to stocks with anomalous returns, the occurrence of a certain event will also attract investor attention and thus influences their behaviors (Kim, 2013; Jiang et al., 2021). Chinese capital market is relatively immature with its high proportion of individual investors (Admati and Pfleiderer, 2009). Strong government intervention is also a distinct feature of the Chinese market economy. Unlike other countries, the Chinese government implements policies more frequently, which introduces greater uncertainty into economic policies (Zhang et al., 2013). As a result, Chinese investors are gradually shifting their attention to policy announcement, which in turn influences their investment decisions (Naseem et al., 2021). Later, it was documented that individual investors begin to pay attention to stock information relating to a financial event after that event occurs and prefer to focus on the market as a whole rather than on a specific stock when faced with a plethora of information (Carro et al., 2015; Balcilar et al., 2017). Concept stocks are the type of stocks that are created as a result of certain events or topics. Investors’ attention to concept stocks has a significant impact on concept stock prices, and accordingly, increased attention can inflate stock prices, boost stock returns, and increase trading volumes.

Policy announcement and investor attention can both have a significant impact on stock markets, but what is the relationship between the three? Some studies argue that investor attention plays a partial role in transferring the impact of industrial policy to overall stock pricing (Baron and Kenny, 1986), while others argue that investor attention moderates the effect for policy announcement (Peng and Xiong, 2006). Through a systematic literature review, we find that although there are abundant studies on the impact of policy announcement or investor attention on stock market, there are few papers that quantitatively analyze the relationship between policy announcement, investor attention, and stock market. Therefore, this paper introduces investor attention into the study of the relationship between policy announcement and stock market, and argues that the promulgation of NEV policies can influence NEV concept stock volatility by attracting investor attention to these stocks. Hereby, we propose the following hypothesis.

*Hypothesis 2*: Investor attention plays a mediating effect in transmitting the impact of new energy vehicle-related policy announcement into new energy vehicle-related concept stock volatility.

## Research Design

### Sample Selection and Data Sources

This paper uses Chinese NEV concept stocks from 2011 to 2020 as the research setting. The stock characteristics data such as opening price, closing price, price fluctuations, market capitalization, total equity, and turnover of 31 NEV concept stocks for 2,294 trading days were obtained from Wind database.

The new energy policy data were collected from the websites of the Ministry of Industry and Information Technology of the People’s Republic of China, the State Council of the People’s Republic of China, the State Taxation Administration of the People’s Republic of China, and the Ministry of Science and Technology of the People’s Republic of China from 2010 and 2020. Baidu index was chosen as a proxy variable for investor attention, and “stock code” was used as a keyword to extract daily Baidu search data using Python scrapping technique. The rest of the control variables were obtained from the China Stock Market and Accounting Research (CSMAR) Database and NetEase Finance website.

### Variables

This section introduces dependent variable, explanatory variable, mediation variable, and control variables, and presents descriptive statistical analysis.

#### Dependent Variable

The volatility of NEV concept stocks (*Vol*) is the dependent variable. Volatility reflects the asset price fluctuations and is an important indicator of uncertainty in return on assets. In this paper, the standard deviation of the log-return of the 20-day moving average is used to represent concept stock volatility (*Vol*), which is calculated as follows.


$$Vol_{i,t}=\sqrt{\frac{\sum {\left(X_{i,t}-\bar{X}\right)}^{2}}{N-1}}$$

$$\bar{X}=\frac{\sum X_{i,t}}{N}$$

Where $Vol_{i,t}$ denotes the volatility of stock *i* in period *t*, *N* is the number of observations, and $X_{i,t}$ represents the logarithmic return of stock *i* in period *t*, which is the natural logarithm of closing price of the day divided by the closing price of the previous day.

#### Explanatory Variable

Policy announcement (*Tep*, *Pap*). Following previous research on NEV-related policies, the national NEV policies are divided into two categories, the PTI, and P&A policies. Following Guo et al. (2020), we construct the dummy variable to quantify the impact of the policy announcement. In the baseline regression, if the policy was announced before the 15th day of the month, the policy is considered to mainly affect the stock market in the current month, and therefore, the dummy variable of the current month is set to 1. If the policy was announced after the 15th day, the policy is considered to mainly affect the stock market in the next month, and therefore, the dummy variable of the next month is set to 1. So, the dummy variable of the remaining months is set to 0. In addition, to test the robustness of the baseline results, this paper reconstructs the dummy variable for policy announcement using the 20th day of the current month as the cut-off point to re-estimate the baseline regression. *Tep* represents the purchase tax incentive policy, and *Pap* represents the P&A policy.

We collected the policies related to NEVs that were issued by government authorities from 2010 to 2020 through the websites of the Ministry of Industry and Information Technology, the State Council, the State Taxation Administration, and the Ministry of Science and Technology of the People’s Republic of China. Because policies issued by local governments are frequently extensions of national policies, only policies issued at the national level are considered for their impact in this paper. PTI policies are implemented to exempt specific types of NEVs from vehicle purchase tax in order to increase the use of NEVs. P&A policies are implemented in each city to promote NEVs, with specific application goals and a variety of government subsidies and incentives. Table 1 summarizes China’s national policies for promoting NEVs from 2010 to 2020, including seven PTI policies and 16 P&A policies.

**Table 1:** List of policies on new energy vehicles from 2010 to 2020.

Date	Policy	Category
30 May 2010	Circular on expanding the demonstration and promotion of energy saving and new energy vehicles in the public service field	P&A
4 June 2010	A circular on pilot subsidies for private buyers of new energy vehicles	P&A
14 October 2011	Circular on further promoting the demonstration and pilot work of energy-saving and new energy vehicles	P&A
6 March 2012	Circular on tax policies for vehicles and vessels using new energy to save energy	PTI
17 September 2013	Notice on continuing the promotion and application of new energy vehicles	P&A
30 September 2013	Notice on the promotion of energy-saving and environment-friendly cars of 1.6 liters or less	P&A
28 January 2014	Notice on further promotion and application of new energy vehicles	P&A
21 July 2014	Guidelines on accelerating the promotion and application of new energy vehicles	P&A
1 August 2014	New energy vehicle purchase tax exemption announcement	PTI
13 March 2015	Implementation opinions of the Ministry of Transport on accelerating the promotion and application of new energy vehicles in the transportation industry	P&A
22 April 2015	Notice on financial Support Policies for the promotion and application of New energy Vehicles from 2016 to 2020	P&A
21 May 2015	Notice on improving the price subsidy policy for refined oil on city buses to speed up the promotion and application of new energy vehicles	P&A
7 May 2015	Circular on preferential tax policies for vehicles and vessels using new energy to save energy	PTI
20 January 2016	Notice on the “13th Five-Year” new energy vehicle charging infrastructure incentive policy and strengthen the promotion and application of new energy vehicles	P&A
29 December 2016	Notice on adjusting fiscal subsidy policies for the promotion and application of new energy vehicles	P&A
26 December 2017	New energy vehicle purchase tax exemption announcement	PTI
12 February 2018	Notice on adjusting and improving fiscal subsidy policies for the promotion and application of new energy vehicles	P&A
10 July 2018	Notice on the preferential policy of vehicle tax for energy-saving and new energy vehicles and vessels	PTI
26 March 2019	Circular on further improving fiscal subsidy policies for the promotion and application of new energy vehicles	P&A
8 May 2019	Notice on supporting the promotion and application of new energy buses	P&A
28 June 2019	Announcement on continued implementation of preferential tax policies for vehicle purchase	PTI
16 April 2020	Announcement on the new energy vehicle purchase tax exemption policy	PTI
23 April 2020	Circular on improving fiscal subsidy policies for the promotion and application of new energy vehicles	P&A

#### Mediation Variable

Investor attention (*Nea*). Following existing studies (Zhang et al., 2013; Zhang and Wang, 2015; Wan et al., 2021), we use Baidu index as a proxy variable and obtain the Baidu index data of the NEV concept stock codes from 4 January 2011 to 10 June 2020 using the Python scrapping technique. The Baidu search index data of 2,294 trading days were eventually retained. Due to the large scale of the Baidu search index data, we take the natural logarithm to mitigate potential heteroskedasticity issues. *Nea* stands for investors’ attention to NEVs.

#### Control Variables

We control for the market capitalization of individual stocks (*Size*), turnover rate (*Tur*), volatility of the CSI 300 index (*Hvol*), stock market crash dummy variable (*Guz*), and first trading day dummy variable (*Time*; Kaplanski and Levy, 2010; Levy and Yagil, 2011). All variables are detailed in Table 2. The descriptive statistics of all variables are displayed in Table 3.

**Table 2:** Variable descriptions.

	Variables	Index	Definition
Dependent variable	*Vol*	New energy vehicle concept stock volatility	Daily volatility of new energy vehicle concept stock from 4 January 2011 to 10 June 2020
Explanatory variable	*Tep*	Purchase tax incentive	National policies related to purchase tax incentives for new energy vehicles from 2011 to 2020
	*Pap*	Promotion and application policy	National policies related to the promotion of new energy vehicles from 2011 to 2020
	*Nea*	Investor attention to new energy vehicle concept stock	Natural logarithm of the sum of Baidu search index of new energy vehicle concept stock codes
Control variable	*Size*	Market capitalization of individual stocks	Natural logarithm of daily market capitalization of individual stocks
	*Tur*	Turnover rate	Ratio of stock turnover to total equity
	*HVol*	Volatility of the CSI 300 index	Daily volatility of the CSI 300 Index from 4 January 2011 to 10 June 2020
	*Guz*	Stock market crash dummy variable	1 for the period from 26 June 2015 to 31 December 2015, and 0 for the rest of the trading days
	*Time*	First trading day dummy variable	1 for the first day of a continuous trading day and 0 for the rest

**Table 3:** Summary statistics.

	Number of observation	Mean	Median	SD
Vol	71,114	0.0260	0.0233	0.0120
Pap	71,114	0.1050	0	0.3070
Tep	71,114	0.0667	0	0.2490
Nea	71,114	6.2443	6.1591	0.6448
Size	71,114	22.6900	22.58	1.0510
Tur	71,114	1.7660	1.123	1.9420
Hvol	71,114	0.0130	0.0116	0.0065
Guz	71,114	0.0558	0	0.2300
Time	71,114	0.2090	0	0.4060

## Results

### Baseline Results

To examine the impact of policy announcement on NEV concept stock volatility, we estimate the following regression:


$$\begin{array}{l} Vol_{i,t}=\alpha_{i}+\beta_{1}Tep+\beta_{2}Pap+\beta_{3}Size_{i,t}+\beta_{4}Tur_{i,t}\\ +\beta_{5}HVol_{i,t}+\beta_{6}Time+\beta_{7}Guz+\gamma_{t}+\mu_{i}+\varepsilon_{i,t} \end{array}$$

where $Vol_{i,t}$ represents the volatility of the *i*th stock in period *t*; *Tep* is purchase tax incentives, and *Pap* is promotion and application policies; and the control variables include market capitalization of individual stocks ( $Size_{i,t}$ ), turnover rate ( $Tur_{i,t}$ ), volatility of the CSI 300 index ( $HVol_{i,t}$ ), first trading day dummy variable ( Time ), and stock market crash variable ( Guz ). We also control for industry fixed effects (*Industry FE*), individual fixed effects (Individual FE), and time fixed effects (Time FE).

For the above panel data model, we conduct the *F*-test, Hausman test, heteroskedasticity test, and cross-sectional dependence test. The results show the presence of individual effects, heteroskedasticity, and cross-sectional dependence. Therefore, this paper adopts the panel corrected standard error (PCSE) method proposed by Beck and Katz (1995) for the empirical analysis, i.e., the standard errors in the fixed-effect model are corrected. To avoid biased results caused by two policies being announced in the same month, we first estimate the baseline model by investigating the impact of purchase tax incentive (*Tep*) and promotion and application policy (*Pap*) on the stock price volatility, respectively; we also add *Tep* and *Pap* together into the baseline model to investigate their respective impact on stock volatility simultaneously. Finally, by comparing the results, the impact of each policy on stock price volatility is clarified. Table 4 displays the results.

**Table 4:** Baseline regression results.

Variables	Model (1)	Model (2)	Model (3)
	Vol	Vol	Vol
Tep	0.00006		−0.00035
	(0.00030)		(0.00031)
Pap		0.00141^***^	0.00147^***^
		(0.00024)	(0.00025)
Size	0.00194^***^	0.00179^***^	0.00179^***^
	(0.00011)	(0.00011)	(0.00011)
Tur	0.00143^***^	0.00141^***^	0.00141^***^
	(0.00002)	(0.00002)	(0.00002)
Hvol	0.96038^***^	0.93860^***^	0.93721^***^
	(0.01374)	(0.01413)	(0.01418)
Guz	0.00869^***^	0.00926^***^	0.00926^***^
	(0.00039)	(0.00040)	(0.00040)
Time	−0.00003	−0.00004	−0.00004
	(0.00018)	(0.00018)	(0.00018)
*Industry FE*	Yes	Yes	Yes
*Individual FE*	Yes	Yes	Yes
*Time FE*	Yes	Yes	Yes
*No. of obs.*	71,114	71,114	71,114
*R-squared*	0.59299	0.59422	0.59426

*** represents significant at the 1% significance level.

Standard deviations are provided in parentheses.

As shown in Table 4, Models (1) and (2) estimate the impact of purchase tax incentives (*Tep*) and *Pap* on the volatility of new energy concept stocks, respectively, by both controlling for market capitalization of individual stocks ( $Size_{i,t}$ ), turnover rate ( $Tur_{i,t}$ ), volatility of the CSI 300 index ( $HVol_{i,t})$ , first trading day dummy variable ( Time ), and stock market crash variable ( Guz ). Both also control for industry, individual, and time fixed effects. The results show that the coefficient of acquisition tax incentives (*Tep*) is positive but not significant, indicating that the acquisition tax incentives does not have an impact on the volatility of NEV concept stocks; however, the coefficient of *Pap* is significantly positive at the 1% significance level, which indicates that the P&A policy has a significant and positive impact on the volatility of NEV concept stocks. Thus, hypotheses H1a and H1b are validated. Model (3) adds both types of policies and control variables, and the results are consistent with the previous results, which proves *Hypothesis 1*. In summary, purchase tax incentives have no significant impact on the volatility of NEV concept stocks, whereas promotion and application policies have a significant and positive impact on the volatility of NEV concept stocks. The volatility of a concept stock depends on the type of policy related to the industry. The purchase tax incentive policy is usually announced at long intervals, with insufficient and minor tax adjustments, and in a unitary manner, making it difficult to influence the NEV market. The promotion and application policy, on the other hand, is targeted at the NEV industry, each new version is an update and supplement to the previous one, and it functions as a market bellwether by indicating the country’s development direction in the field. Therefore, it exerts a significant impact on the NEV concept stock market.

### The Mediating Effect of Investor Attention

NEV promotion and application policies have a significant and positive on the volatility of NEV concept stocks, while purchase tax incentive policies have no significant effect, so we further study the promotion and application policy in the followings. Investors have a limited ability to allocate attention and interpret information, and policy announcement will further affect how investors allocate attention to specific events and information. In this case, we hypothesize that promotion and application policies may increase the volatility of NEV concept stocks by increasing investors’ attention to NEV concept stocks. In order to test this hypothesis, we use Python scrapping technique to extract the Baidu search index of different NEV concept stocks with their stock code as the keyword. We take the log of the sum of Baidu search index of NEV concept stock codes to calculate investor attention and investigate the mediating effect of investor attention.

As shown in Table 5, the coefficient of *Pap* is significantly positive at the 1% significance level in Model (1), and the positive significance holds in Model (2) where the dependent variable is *Nea* (investor attention). Meanwhile, the coefficients of *Pap* and *Nea* are both significantly positive at the 1% significance level in Model (3) which adds both *Pap* and *Nea* into the model. This indicates that NEV promotion and application policies not only raise the volatility of NEV concept stocks directly but also increase the volatility through boosting investor attention. For accuracy purposes, we conduct a Sobel test on the mediation model with *a* = 0.12971, *b* = 0.00155, $S_{a}$ = 0.02037, and $S_{b}$ = 0.00015, and the results show that the test value of p is much less than 0.01. Therefore, investor attention partially mediates the impact of promotion and application policy announcement on the volatility of NEV concept stocks proving *Hypothesis 2*.

**Table 5:** The mediating effect of investor attention.

Variables	Model (1)	Model (2)	Model (3)
	Vol	Nea	Vol
Pap	0.00141^***^	0.12971^***^	0.00121^***^
	(0.00024)	(0.02037)	(0.00025)
Nea			0.00155^***^
			(0.00015)
Size	0.00179^***^	0.03239^***^	0.00174^***^
	(0.00011)	(0.00808)	(0.00011)
Tur	0.00141^***^	0.11026^***^	0.00123^***^
	(0.00002)	(0.00146)	(0.00003)
Hvol	0.93860^***^	9.74427^***^	0.92348^***^
	(0.01413)	(1.17959)	(0.01450)
Guz	0.00926^***^	0.26469^***^	0.00884^***^
	(0.00040)	(0.03335)	(0.00041)
Time	−0.00004	0.01026	−0.00006
	(0.00018)	(0.01516)	(0.00019)
*Industry FE*	Yes	Yes	Yes
*Individual FE*	Yes	Yes	Yes
*Time FE*	Yes	Yes	Yes
*No. of obs.*	71,114	71,114	71,114
*R-squared*	0.59422	0.60746	0.59693

*** represents significant at the 1% significance level.

Standard deviations are provided in parentheses.

### Heterogeneity Analysis

Given differences in the equity ownership of NEV concept stocks, the relationship between policy announcement, investor attention, and NEV concept stocks may vary depending on the equity ownership. Therefore, based on the equity ownership information provided by the CSMAR database, we categorize the sample data into state-owned enterprises and non-state-owned enterprises (Su et al., 2020). We introduce the dummy variable *SOE*, which is assigned to 1 if the stock belongs to a state-owned enterprise and 0 if it belongs to a non-state-owned enterprises. The results are shown in Table 6, respectively.

**Table 6:** Heterogeneity results for state ownership.

Variables	SOEs			Non-SOEs		
	Model (1)	Model (2)	Model (3)	Model (4)	Model (5)	Model (6)
	Vol	Vol	Vol	Vol	Vol	Vol
Tep	−0.00007		−0.00040	0.00026		−0.00013
	(0.00030)		(0.00031)	(0.00034)		(0.00035)
Pap		0.00106^***^	0.00114^***^		0.00131^***^	0.00134^***^
		(0.00025)	(0.00026)		(0.00028)	(0.00029)
Size	0.00193^***^	0.00182^***^	0.00183^***^	0.00196^***^	0.00185^***^	0.00186^***^
	(0.00014)	(0.00014)	(0.00014)	(0.00011)	(0.00011)	(0.00011)
Tur	0.00135^***^	0.00133^***^	0.00132^***^	0.00149^***^	0.00148^***^	0.00148^***^
	(0.00003)	(0.00003)	(0.00003)	(0.00003)	(0.00003)	(0.00003)
Hvol	0.98996^***^	0.97974^***^	0.97849^***^	0.92223^***^	0.90718^***^	0.90677^***^
	(0.01384)	(0.01401)	(0.01404)	(0.01552)	(0.01574)	(0.01578)
Guz	0.00847^***^	0.00880^***^	0.00880^***^	0.00902^***^	0.00943^***^	0.00943^***^
	(0.00039)	(0.00040)	(0.00040)	(0.00045)	(0.00045)	(0.00045)
Time	−0.00006	−0.00007	−0.00007	0.00002	0.00000	0.00000
	(0.00018)	(0.00018)	(0.00018)	(0.00021)	(0.00021)	(0.00021)
*Industry FE*	Yes	Yes	Yes	Yes	Yes	Yes
*Individual FE*	Yes	Yes	Yes	Yes	Yes	Yes
*Time FE*	Yes	Yes	Yes	Yes	Yes	Yes
*No. of obs.*	38,998	38,998	38,998	32,116	32,116	32,116
*R-squared*	0.60771	0.60895	0.60900	0.57065	0.57196	0.57201

*** represents significant at the 1% significance level.

Standard deviations are provided in parentheses.

As shown in Table 6, the coefficients of *Tep* are not significant in Model (1) and (4), indicating no significant impact of the acquisition tax incentive policy on the volatility of NEV concept stocks regardless of whether the firm is state-owned or not. As indicated by the results of Model (2) and (5), although the announcement of the P&A policy has a significant and positive effect on the stock volatility of state-owned and non-state-owned enterprises, the coefficient difference indicates heterogeneity in the state ownership (value of p of test statistic <0.01). Compared with state-owned enterprises, non-state-owned enterprises are more sensitive to NEV promotion and application policies, indicating that when a promotion and application policy was implemented, non-state-owned enterprises are more willing to invest in innovation and energy transition, which in turn increases their stock volatility risk. The results of Model (3) and (6) further validate this finding by indicating that the stock price behavior of NEV concept stocks of state-owned enterprises is more stable than that of non-state-owned enterprises and that state-owned enterprises are able to reduce the risk of volatility caused by investor attention through self-regulation.

### Robustness Test

To further test the robustness of the baseline results, we adopt two methods—alternative measure and random sample selection—to verify the impact of policy announcement on the volatility of NEV concept stocks.

#### Alternative Measure

In the baseline regression, we construct two dummy variables, *Tep* and *Pap,* using the 15th day of the month as the cut-off point. In this section, to avoid the randomness of regression results caused by the 15th day cut-off, we reconstruct the dummy variables *Tep1* and *Pap1* for the NEV policy using the 20th day of the month as the cut-off point instead. The regression results in Table 7 show that the acquisition tax incentive policy has no significant impact on stock volatility, whereas the P&A policy has a significant and positive effect on the volatility of NEV concept stocks, demonstrating the robustness of the empirical results.

**Table 7:** Robustness checks for the alternative measure.

Variables	Model (1)	Model (2)	Model (3)
	Vol	Vol	Vol
Tep1	0.00003		−0.00012
	(0.00030)		(0.00030)
Pap1		0.00107^***^	0.00108^***^
		(0.00024)	(0.00024)
Size	0.00194^***^	0.00186^***^	0.00186^***^
	(0.00011)	(0.00011)	(0.00011)
Tur	0.00143^***^	0.00141^***^	0.00141^***^
	(0.00002)	(0.00002)	(0.00002)
Hvol	0.96028^***^	0.94944^***^	0.94945^***^
	(0.01374)	(0.01389)	(0.01389)
Guz	0.00868^***^	0.00902^***^	0.00901^***^
	(0.00039)	(0.00040)	(0.00040)
Time	−0.00003	−0.00004	−0.00004
	(0.00018)	(0.00018)	(0.00018)
*Industry FE*	Yes	Yes	Yes
*Individual FE*	Yes	Yes	Yes
*Time FE*	Yes	Yes	Yes
*No. of obs.*	71,114	71,114	71,114
*R-squared*	0.59299	0.59374	0.59374

*** represents significant at the 1% significance level.

Standard deviations are provided in parentheses.

#### Random Sample Selection

To ensure the applicability of the results, we randomly select 10 stocks from the 31 NEV concept stocks to re-estimate the baseline regression. Considering that the total sample contains six types of NEV stocks, such as computer communication and other electronic equipment manufacturing, automobile manufacturing, rubber and plastic product manufacturing, and so on, we stratified random sampling by industry type. In each sampling stratum, direct random sampling of the stock codes was conducted using the “*Sample*” command in the Stata software, and the appropriate number of stocks was selected from each stratum according to the proportion of stocks in different industry types in the total sample. After stratified sampling, 10 stock codes were obtained, and the final randomly selected sample was formed by matching the relevant stock trading data from 2011 to 2020 to the stock codes. The 10 randomly selected stocks include Shenzhen Kaifa Technology Co., Ltd. (000021), Weichai Power Co., Ltd. (000338), Guizhou Tire Co., Ltd. (000589), Zhongtong Bus Co., Ltd. (000957), Guoguang Electric Co., Ltd. (002045), Shenzhen Auto Electric Power Plant Co., Ltd. (002227), Shenzhen INVT Electric Co., Ltd. (002334), Chengdu Xinzhu Road and Bridge Machinery Co., Ltd. (002480), SAIC Motor Co., Ltd. (600104), and Neusoft Corp. (600718). These 10 concept stocks contain all the NEV stock types from the total sample data, which eliminates the estimation bias arising from the sampling restricted to certain industries and ensures generalizability and applicability of the randomly selected sample, making the results more reliable. We re-estimate the fixed-effect model with robust standard errors on the randomly selected sample. The results, as shown in Table 8, are consistent with the full-sample results, again indicating that the baseline results are robust and reliable.

**Table 8:** Robustness checks for random sampling.

Variables	Model (1)	Model (2)	Model (3)
	Vol	Vol	Vol
Tep	−0.00013		−0.00044
	(0.00038)		(0.00038)
Pap		0.00104^***^	0.00113^***^
		(0.00031)	(0.00032)
Size	0.00349^***^	0.00334^***^	0.00335^***^
	(0.00020)	(0.00020)	(0.00020)
Tur	0.00154^***^	0.00153^***^	0.00153^***^
	(0.00003)	(0.00003)	(0.00003)
Hvol	0.97608^***^	0.96002^***^	0.95830^***^
	(0.01726)	(0.01781)	(0.01788)
Guz	0.00792^***^	0.00836^***^	0.00836^***^
	(0.00049)	(0.00050)	(0.00050)
Time	0.00002	0.00001	0.00001
	(0.00023)	(0.00023)	(0.00023)
*Industry FE*	Yes	Yes	Yes
*Individual FE*	Yes	Yes	Yes
*Time FE*	Yes	Yes	Yes
*No. of obs.*	22,940	22,940	22,940
*R-squared*	0.58406	0.58466	0.58473

*** represents significant at the 1% significance level.

Standard deviations are provided in parentheses.

## Conclusion

The paper takes NEV concept stocks as the research setting and selects NEV-related policies from 2011 to 2020 to examine the relationship between the volatility behavior of NEV concept stocks and relevant policy announcement including NEV acquisition tax incentives and promotion and application policies. We also explore the mediating effect of investor attention in the relationship between policy announcement and NEV concept stocks. We find that different types of policies have different impacts on NEV concept stocks. The PTI policy does not have a significant impact on the volatility of NEV concept stocks due to their long promulgation interval, insignificant content adjustments, and unitary form, whereas the P&A policy has a significant and positive impact on the volatility of the NEV concept stocks, in which investor attention plays a partial mediating effect. The findings indicate that the introduction of NEV P&A policies has brought about effects to the stock market while also attracting a lot of investor attention. In addition, state-owned NEV firms are less affected by new policies than non-state-owned firms, as they can self-regulate to mitigate the volatility risk brought on by investor attention.

In order to achieve green economic recovery in the post-pandemic era, governments should improve the supporting policies for the new energy industry. Based on our findings, we propose the following recommendations. First, the purpose of NEV policies is to promote energy transition in China’s auto industry and achieve low-carbon economic recovery. However, because China’s NEV industry has been in the early development stage for the past 10 years and has a lot of room for improvement, the reactions of the society and individuals should be considered comprehensively when formulating policies. Moreover, the government should prioritize NEV promotion and application policies over purchase tax incentive policies, and relevant policies should be updated as the market develops to maintain the policy’s effectiveness, stability, and continuity.

Second, the impact of investor attention on the stock market should not be overlooked. The government should encourage rational stock investment and provide guidance to individual investors in identifying risks and making sensible investment decisions. Moreover, the government should improve corporate operation regulations, increase stock market transparency, provide symmetric information to investors, and monitor concept stocks.

Third, the government should vigorously support the development of NEV-related industries, including state-owned firms or non-state-owned firms, while closely monitoring the effectiveness of policies for state-owned NEV firms. In addition, policymakers should consider different firm ownerships when formulating policies and improve the risk control ability of non-state-owned NEV firms when revising policies. At the same time, the government should encourage state-owned NEV firms to participate in energy transition and enhance their competitiveness.

## Data Availability Statement

The original contributions presented in the study are included in the article/supplementary material, further inquiries can be directed to the corresponding author.

## Author Contributions

MS: conceptualization, investigation, validation, and writing—review and editing. CW: formal analysis, methodology, and writing—original draft. All authors contributed to the article and approved the submitted version.

## Funding

This research is supported by the Social Science Planning Project of Shandong Province (grant number: 19CDN26).

## Conflict of Interest

The authors declare that the research was conducted in the absence of any commercial or financial relationships that could be construed as a potential conflict of interest.

## Publisher’s Note

All claims expressed in this article are solely those of the authors and do not necessarily represent those of their affiliated organizations, or those of the publisher, the editors and the reviewers. Any product that may be evaluated in this article, or claim that may be made by its manufacturer, is not guaranteed or endorsed by the publisher.

## Footnotes

^1^The policy is as: “*Notice on Improving the Fiscal Subsidy Policy for the Promotion and Application of New Energy Vehicles,*” from the Chinese government website: http://www.gov.cn/

^2^The information is sourced from the official website of Baidu Index: https://index.baidu.com/v2/index.html#/

## References

[ref1] Aboody D. Lehavy R. Trueman B. (2010). Limited attention and the earnings announcement returns of past stock market winners. Rev. Acc. Stud. 15, 317–344. doi: 10.1007/s11142-009-9104-9

[ref2] Admati A. R. Pfleiderer P. (2009). The “wall street walk” and shareholder activism: exit as a form of voice. Rev. Financ. Stud. 22, 2645–2685. doi: 10.1093/rfs/hhp037

[ref3] Aouadi A. Arouri M. Roubaud D. (2018). Information demand and stock market liquidity: international evidence. Econ. Model. 70, 194–202. doi: 10.1016/j.econmod.2017.11.005

[ref4] Balcilar M. Demirer R. Ulussever T. (2017). Does speculation in the oil market drive investor herding in emerging stock markets? Energy Econ. 65, 50–63. doi: 10.1016/j.eneco.2017.04.031

[ref5] Barber B. M. Odean T. (2008). All that glitters: The effect of attention and news on the buying behavior of individual and institutional investors. Rev. Financ. Stud. 21, 785–818. doi: 10.1093/rfs/hhm079

[ref7] Barberis N. Thaler R. (2005). A Survey of Behavioral Finance. United States: Princeton University Press, 1–76.

[ref8] Baron R. M. Kenny D. A. (1986). The moderator–mediator variable distinction in social psychological research: conceptual, strategic, and statistical considerations. J. Pers. Soc. Psychol. 51, 1173–1182. doi: 10.1037/0022-3514.51.6.1173, PMID: 3806354

[ref9] Beck N. Katz J. N. (1995). What to do (and not to do) with time-series cross-section data. Am. Polit. Sci. Rev. 89, 634–647. doi: 10.2307/2082979

[ref10] Carro A. Toral R. San Miguel M. (2015). Markets, herding and response to external information. PLoS One 10:e0133287. doi: 10.1371/journal.pone.0133287, PMID: 26204451 PMC4512694

[ref11] Chen S. T. Haga K. Y. A. (2021). Using E-GARCH to analyze the impact of investor sentiment on stock returns near stock market crashes. Front. Psychol. 12:664849. doi: 10.3389/fpsyg.2021.664849 34385951 PMC8354526

[ref12] Cooper J. C. Kovacic W. E. (2012). Behavioral economics: implications for regulatory behavior. J. Regul. Econ. 41, 41–58. doi: 10.1007/s11149-011-9180-1

[ref13] Engle R. F. Rangel J. G. (2008). The spline-GARCH model for low-frequency volatility and its global macroeconomic causes. Rev. Financ. Stud. 21, 1187–1222. doi: 10.1093/rfs/hhn004

[ref14] Fang L. Peress J. (2009). Media coverage and the cross-section of stock returns. J. Financ. 64, 2023–2052. doi: 10.1111/j.1540-6261.2009.01493.x

[ref15] Foresti P. Napolitano O. (2017). On the stock market reactions to fiscal policies. Int. J. Financ. Eco. 22, 296–303. doi: 10.1002/ijfe.1584

[ref16] Guo M. Kuai Y. Liu X. (2020). Stock market response to environmental policies: evidence from heavily polluting firms in China. Econ. Model. 86, 306–316. doi: 10.1016/j.econmod.2019.09.028

[ref17] He P. Nie G. Wang G. Zhang X. (2011). Optimal monetary policy in China. Chin. World. Econ. 19, 83–105. doi: 10.1111/j.1749-124X.2011.01228.x

[ref18] Hirshleifer D. Teoh S. H. (2003). Limited attention, information disclosure, and financial reporting. J. Account. Econ. 36, 337–386. doi: 10.1016/j.jacceco.2003.10.002

[ref19] Hsiao C. Y. L. Wei X. Sheng N. Shao C. (2021). A joint test of policy contagion with application to the solar sector. Renew. Sust. Energ. Rev. 141:110762. doi: 10.1016/j.rser.2021.110762

[ref20] Jiang B. Zhu H. Zhang J. Yan C. Shen R. (2021). Investor sentiment and stock returns during the COVID-19 pandemic. Front. Psychol. 12:708537. doi: 10.3389/fpsyg.2021.708537, PMID: 34354650 PMC8329237

[ref21] Kaplanski G. Levy H. (2010). Exploitable predictable irrationality: The FIFA world cup effect on the US stock market. J. Financ. Quant. Anal. 45, 535–553. doi: 10.1017/S0022109010000153

[ref22] Kim Y. H. A. (2013). Self attribution bias of the CEO: evidence from CEO interviews on CNBC. J. Bank. Financ. 37, 2472–2489. doi: 10.1016/j.jbankfin.2013.02.008

[ref23] Levy T. Yagil J. (2011). Air pollution and stock returns in the US. J. Econ. Psychol. 32, 374–383. doi: 10.1016/j.joep.2011.01.004

[ref24] Liu W. Zeng L. Wang Q. (2021). Psychological distance toward air pollution and purchase intention for new energy vehicles: An investigation in China. Front. Psychol. 12:569115. doi: 10.3389/fpsyg.2021.569115 33868068 PMC8046919

[ref25] Loh R. K. (2010). Investor inattention and the underreaction to stock recommendations. Financ. Manag. 39, 1223–1252. doi: 10.1111/j.1755-053X.2010.01110.x

[ref26] Naseem S. Mohsin M. Hui W. Liyan G. Penglai K. (2021). The investor psychology and stock market behavior during the initial era of COVID-19: a study of China, Japan, and the United States. Front. Psychol. 12:626934. doi: 10.3389/fpsyg.2021.626934 33643158 PMC7902781

[ref27] Pastor L. Veronesi P. (2012). Uncertainty about government policy and stock prices. J. Financ. 67, 1219–1264. doi: 10.1111/j.1540-6261.2012.01746.x

[ref28] Peng L. Xiong W. (2006). Investor attention, overconfidence and category learning. J. Financ. Econ. 80, 563–602. doi: 10.1016/j.jfineco.2005.05.003

[ref29] Poon S. Taylor S. J. (1991). Macroeconomic factors and the UK stock market. J. Bus. Financ. Acc. 18, 619–636. doi: 10.1111/j.1468-5957.1991.tb00229.x

[ref30] Ramiah V. Martin B. Moosa I. (2013). How does the stock market react to the announcement of green policies? J. Bank. Financ. 37, 1747–1758. doi: 10.1016/j.jbankfin.2013.01.012

[ref31] Sam A. G. Zhang X. (2020). Value relevance of the new environmental enforcement regime in China. J. Corp. Finan. 62:101573. doi: 10.1016/j.jcorpfin.2020.101573

[ref32] Seasholes M. S. Wu G. (2007). Predictable behavior, profits, and attention. J. Empir. Financ. 14, 590–610. doi: 10.1016/j.jempfin.2007.03.002

[ref33] Simon H. A. (1955). A behavioral model of rational choice. Q. J. Econ. 69, 99–118. doi: 10.2307/1884852

[ref34] Su X. Zhou S. Xue R. Tian J. (2020). Does economic policy uncertainty raise corporate precautionary cash holdings? Acc. Financ. 60, 4567–4592. doi: 10.1111/acfi.12674

[ref35] Tian J. Pan C. Xue R. Yang X. Wang C. Ji X.. (2020). Corporate innovation and environmental investment: The moderating role of institutional environment. Adv. Clim. Chang. Res. 11, 85–91. doi: 10.1016/j.accre.2020.05.003

[ref36] Tian J. Yu L. Xue R. Zhuang S. Shan Y. (2021). Global low-carbon energy transition in the post-COVID-19 era. Appl. Energy 307:118205. doi: 10.1016/j.apenergy.2021.118205 34840400 PMC8610812

[ref37] Tversky A. Kahneman D. (1973). Availability: A heuristic for judging frequency and probability. Cogn. Psychol. 5, 207–232. doi: 10.1016/0010-0285(73)90033-9

[ref38] Wan D. Xue R. Linnenluecke M. Tian J. Shan Y. (2021). The impact of investor attention during COVID-19 on investment in clean energy versus fossil fuel firms. Financ. Res. Lett. 43:101955. doi: 10.1016/j.frl.2021.101955 PMC9665964 36406287

[ref39] Wu Z. Shao Q. Su Y. Zhang D. (2021). A socio-technical transition path for new energy vehicles in China: A multi-level perspective. Technol. Forecast. Soc. Chang. 172:121007. doi: 10.1016/j.techfore.2021.121007

[ref40] Xue R. Qian G. Qian Z. Li L. (2019). Environmental turmoil and firms’ core structure dynamism: The moderating role of strategic alliances. J. Bus. Ind. Market. 34, 1619–1638. doi: 10.1108/JBIM-11-2018-0330

[ref41] Xue R. Qian G. Qian Z. Li L. (2021). Entrepreneurs’ implicit and explicit achievement motives and their early international commitment. Manag. Int. Rev. 61, 91–121. doi: 10.1007/s11575-020-00436-5

[ref42] Yang Y. Xue R. Yang D. (2020). Does market segmentation necessarily discourage energy efficiency? PLoS One 15:e0233061. doi: 10.1371/journal.pone.0233061, PMID: 32437475 PMC7241797

[ref43] You W. Guo Y. Zhu H. Tang Y. (2017). Oil price shocks, economic policy uncertainty and industry stock returns in China: asymmetric effects with quantile regression. Energy Econ. 68, 1–18. doi: 10.1016/j.eneco.2017.09.007

[ref44] Zhang L. Chen W. Hu N. (2021a). Economic policy uncertainty and stock liquidity: evidence from China. Int. J. Emerg. Mark. doi: 10.1108/IJOEM-06-2020-0625 [Epub ahead of print]

[ref45] Zhang W. Li B. Xue R. Wang C. Cao W. (2021b). A systematic bibliometric review of clean energy transition: implications for low-carbon development. PLoS One 16:e0261091. doi: 10.1371/journal.pone.0261091, PMID: 34860855 PMC8641874

[ref46] Zhang W. Shen D. Zhang Y. Xiong X. (2013). Open source information, investor attention, and asset pricing. Econ. Model. 33, 613–619. doi: 10.1016/j.econmod.2013.03.018

[ref47] Zhang B. Wang Y. (2015). Limited attention of individual investors and stock performance: evidence from the ChiNext market. Econ. Model. 50, 94–104. doi: 10.1016/j.econmod.2015.06.009

[ref48] Zhu X. Li R. (2017). An analysis of decoupling and influencing factors of carbon emissions from the transportation sector in the Beijing-Tianjin-Hebei area, China. Sustainability 9:722. doi: 10.3390/su9050722

